# Editorial: Improving surgical outcomes after pancreatic resection

**DOI:** 10.3389/fonc.2022.1024928

**Published:** 2022-11-25

**Authors:** Damiano Caputo, Mark Girgis

**Affiliations:** ^1^ Department of Surgery and Research Unit of General Surgery, Università Campus Bio-Medico di Roma, Rome, Italy; ^2^ Operative Research Unit of General Surgery Unit, Fondazione Policlinico Universitario Campus Bio-Medico, Rome, Italy; ^3^ Department of Surgery, UCLA David Geffen School of Medicine, Los Angeles, CA, United States

**Keywords:** pancreatic surgery outcomes, pancreatoduodenectomy, distal pancreatectomy, laparoscopic surgery, neoadjuvant, nanotechnology, omic analyses, early diagnosis

## Introduction

Surgical resection still represents the most important therapy for pancreatic cancer and periampullary tumors. Unfortunately, surgery is precluded in the majority of cases at the moment of their first detection. Nonetheless, despite recent advances, pancreatic surgery is still burdened with high rates of postoperative morbidity and significant mortality, even in high-volume centers. Furthermore, the heaviness of the surgical procedures and the postoperative quality of life implications resulting from the decreased pancreatic function after surgery often have an impact on long-term outcomes (1).

Indeed, improving outcomes for patients undergoing pancreatic surgery represents an urgent task. To tackle this issue, refining interventions in pre-surgical, intraoperative, and post-surgical care are needed.

The aim of this Research Topic is to bring together all relevant research in the field to provide pancreatic cancer and periampullary tumors care teams with new options for treating and curing patients undergoing surgery or refinements on existing procedures and pathways of care provided to patients.

## Pre-surgical phase

Early diagnosis, careful selection of patients for surgery or neoadjuvant therapy, and optimization of clinical conditions are essential for the successful surgical management of pancreatic and periampullary tumors (2–4).

Usually, the prognosis of these tumors remains poor even after resection because of their late detection. Due to the lack of reliable biomarkers to be used for timely diagnosis, and thanks to recent technological advancements in the fields of “omics” and nanotechnologies, there is growing evidence supporting the development of new strategies for early diagnosis that in the near future could represent a turning point in the fight against these dreadful tumors (5, 6).

Moreover, due to the poor long-term outcomes and the high rates of morbidity and mortality of pancreatic surgery, it would be useful to identify patients who can benefit from invasive and life-threatening surgeries. Different preoperative features have been associated with outcomes, but many are not easy to assess before surgery. To overcome this limit, and aim to develop a reproducible and reliable tool, Zhang et al. analyzes a series of 438 patients who received pancreatic surgery for pancreatic ductal adenocarcinoma (PDAC) and constructs a six-variable nomogram able to predict the prognosis based on common preoperative serum markers such as the neutrophil-to-lymphocyte ratio (NLR), platelet-to-lymphocyte ratio (PLR), Ca 19.9, etc.

A careful selection of patients who can benefit from upfront surgery is even more needed because of the promising data supporting the benefit of neoadjuvant therapies. However, in this scenario, it should be pointed out, as done by He et al., that for patients affected by locally advanced tumors after neoadjuvant therapies, alternatives to surgical resection such as electroporation are proving to offer similar survival results with significantly lower rates of complications.

In the pre-surgical optimization of patients affected by pancreatic cancer and periampullary tumors, jaundice management still represents a relevant and debated aspect. Many studies have reported a significant reduction in postoperative complications in jaundiced patients who underwent surgery without receiving preoperative biliary drainage. Nonetheless, Pande et al. has shown that a direct-to-surgery approach without preoperative biliary drainage may also be associated with longer survival after surgery.

## Intraoperative phase

The main purpose of surgical resection is the complete excision of the disease with the achievement of clear resection margins (R0 resection). In the achievement of this goal, during the surgery, the pathologist supports the pancreatic surgeon. As highlighted by Chen et al., although without a direct impact on survival, the definition of the state of the margins at the frozen section examination is mandatory for the achievement of R0.

Postoperative Pancreatic Fistula (POPF) represents the Achilles’ heel of pancreatic surgery and when clinically relevant (CR-POPF) may lead to other, even lethal, complications mainly in patients who underwent pancreaticoduodenectomy (PD). Historically, the struggle of pancreatic surgeons against this fearful complication starts during the surgery, at the moment of the management of the pancreatic stump. Despite the countless different reconstruction strategies proposed and compared over the years, to date, there is still no evidence that one technique is superior to another (2).

Cao et al. conducts a systematic review and a metanalysis on 1100 PDs and found that invagination techniques may provide a low rate of CR-POPF in presence of a soft stump. However, the author did not report any positive effect on the length of stay or mortality.

Advancements in surgical technologies and equipment boosted the application of the laparoscopic approach to challenging procedures such as PD even in elderly patients. As demonstrated in the systematic review by Wang et al., while considering the limitations of the surgeon’s skills, learning curve, and absence of prospective randomized studies, laparoscopic PD could be considered safe even for the elderly and may benefit from a shorter hospital stay.

It might seem paradoxical, but despite these recent technological advances, one of the open questions in pancreatic surgery still is related to the utility of routine use of abdominal drains (7).

Perhaps, as demonstrated in the recent meta-analysis conducted by Liu et al., this age-old question cannot be solved with a straightforward “yes” or “no”. Some PD patients, especially those with high pancreatic fistula scores may benefit from drains placement, while the current evidence would seem to discourage the routine use of drains for distal pancreatectomies (DPs). However, the many limitations of the available studies do not allow one to draw clear conclusions and further larger randomized studies are still needed on this topic.

## Post-surgical phase

After the surgery and mostly after pancreaticoduodenectomy (PD), delayed gastric emptying (DGE) affects a significant number of patients. Much of the evidence is in favor of early oral feeding after PD, but there are few randomized trials focused on this topic. Researchers of The First Affiliated Hospital of Nanjing Medical University conduct a prospective-randomized controlled trial to test if early oral feeding (EOF), adopted by the majority of pancreatic surgeons, could really reduce DGE compared with early enteral feeding, recommended by the European Society for Parenteral and Enteral Nutrition (ESPEN) after gastrointestinal surgery. The analysis confirms the safety and efficacy of EOF and highlights how additional enteral feeding is not always necessary after PD.

## Conclusions

In order to improve pancreatic surgery outcomes, further studies are required to define tools for early cancer detection, optimize preoperative pathways, carefully select patients for surgery, and reduce postoperative complications.

## Author contributions

The authors confirm being the sole contributor of this work and has approved it for publication.

## Conflict of interest

The authors declare that the research was conducted in the absence of any commercial or financial relationships that could be construed as a potential conflict of interest.

## Publisher’s note

All claims expressed in this article are solely those of the authors and do not necessarily represent those of their affiliated organizations, or those of the publisher, the editors and the reviewers. Any product that may be evaluated in this article, or claim that may be made by its manufacturer, is not guaranteed or endorsed by the publisher.
